# Endoscopic Submucosal Dissection for Esophageal Cancer: Current and Future

**DOI:** 10.3390/life13040892

**Published:** 2023-03-27

**Authors:** Yuki Okubo, Ryu Ishihara

**Affiliations:** Department of Gastrointestinal Oncology, Osaka International Cancer Institute, 1-69 Otemae 3-chome, Chuo-ku, Osaka 541-8562, Japan

**Keywords:** esophageal cancer, endoscopic submucosal dissection, endoscopic mucosal resection, squamous cell carcinoma, Barrett’s adenocarcinoma, complication

## Abstract

Endoscopic submucosal dissection (ESD) has been widely used to treat superficial esophageal cancer. The advantages of esophageal ESD include a high en bloc resection rate and accurate pathological diagnosis. It enables local resection of the primary tumor and accurate identification of the risk factors for lymph node metastasis, including depth, vascular invasion, and types of invasion. Even in cases with clinical T1b-SM cancer, ESD and additional treatment can achieve radical cure, depending on the risk of lymph node metastasis. Esophageal ESD will be increasingly vital in minimally invasive and effective esophageal cancer treatment. This article describes the current status and prospects of esophageal ESD.

## 1. Introduction

Esophageal cancer is one of the most aggressive cancers, with a high mortality rate and poor prognosis. It is the seventh-most-common cancer and the sixth leading cause of cancer death globally, with 604,100 incidences and 544,076 deaths in 2020 [1]. Treatments for advanced esophageal cancer, including surgery and chemoradiotherapy, are invasive, and the prognosis is poor. Brownish area by narrow-band imaging (NBI) has been reported to have excellent detection and diagnostic accuracy for esophageal squamous neoplasia and superficial squamous cell carcinoma (SCC) [2]. The dot-like vessels and background coloration (BGC) are useful findings in diagnosing these lesions [3]. Although iodine staining has a high sensitivity but low specificity for detecting SCC, the pink color sign (recognized as pink 2–3 min after staining) has been reported to be useful in differentiating between cancer and noncancer lesions (a sensitivity of 88% and specificity of 95%) [4]. Owing to these reports, the detection rate of esophageal cancer has been improving, and many lesions are now being treated endoscopically. In Japan, endoscopic submucosal dissection (ESD) for superficial esophageal cancer is the mainstay of endoscopic treatment for superficial esophageal cancer, with overall en bloc resection and local recurrence rates, reportedly, better than endoscopic mucosal resection (EMR) [5]. Though ESD is a minimally invasive treatment, it is associated with the risk of complications, and appropriate judgment is required to determine its indications. Therefore, we reviewed the general knowledge that should be noted to perform esophageal ESD safely.

## 2. ESD Indications for SCC

The indication for ESD in patients with esophageal SCC is determined mainly based on cancer invasion depth and the lateral extent of the cancer. The Japanese EMR/ESD guidelines suggested the indication for ESD as follows (Table 1) [6].

### 2.1. Cancer Invasion Depth

Clinical epithelial/lamina propria mucosae (EP/LPM) esophageal cancer is considered a good indication for ESD because pathological EP/LPM lesions have an extremely low risk of lymph node metastasis and can be cured by endoscopic resection (ER) [7,8,9,10]. The treatment indication of clinical muscularis mucosae or submucosa 1 (MM/SM1) cancer lesions is crucial. The depth of the lesion is usually diagnosed by non-magnifying and magnifying endoscopy. The use of EUS is controversial. Preoperative diagnosis of cancer invasion depth for MM/SM1 cancer is not accurate enough. For example, 27.4% and 55.2% of clinical MM/SM1 cancers diagnosed by magnifying NBI and endoscopic ultrasound (EUS), respectively, were pathological EP/LPM cancer [8,9,10,11,12,13]. Considering preoperative diagnosis accuracy, the diagnostic ESD may be justified as the initial treatment for clinical MM/SM1 cancer. Thus, the guideline states that “ER is weakly recommended as initial treatment for esophageal SCC with a clinical T1a-MM/T1b-SM1 cancer.” However, the need for adjuvant therapy should be determined based on the histologic findings of the resected specimen.

### 2.2. Lateral Extent of Cancer

Although ESD is an effective treatment, extensive esophageal ER may cause postoperative esophageal stricture. The incidence of postoperative stricture is 60.7–75% for 3/4 to semi-circumferential resection and 100% for whole circumferential resection without any prophylactic measures. Stenosis after esophageal ESD can cause dysphagia, necessitating multiple endoscopic balloon dilatations. It negatively impacts the quality of life (QOL) and delays additional chemoradiotherapy. However, the stenosis rate after non-circumferential resection with appropriate prophylaxis can be reduced to 11.3–36.2%. Accordingly, noncircumferential lesions are considered an indication of ESD. As for circumferential lesions, the risk of stenosis after resection remains high, even with preventive measures. A long diameter of 50 mm or greater has been reported as a risk for stenosis [14]. In addition, for cMM/SM1 lesions, the greater the circumference, the higher the possibility of pMM/SM1 or deeper, with 86% of whole circumferential cMM/SM1 lesions reported being deeper than pMM/SM1. Thus, ESD is not recommended for whole circumferential cMM/SM1 lesions because of the high risk of stenosis and the possibility of delaying additional treatment [15].

## 3. Treatment Outcomes

### 3.1. ESD

The ESD procedure for esophageal cancer is generally more complex than gastric ESD because of the following anatomical characteristics of the esophagus: (1) it has a thin muscular layer, with no serous membrane; (2) it has a narrow lumen and compressions of the aorta, vertebral body, and trachea; (3) it is easily affected by heartbeat, respiratory movements, and contraction of the internal ring muscle; and (4) it lies in the posterior mediastinum. In addition, because of surrounding organs including the lungs, there is a risk of pneumothorax or mediastinitis in the case of perforation. However, in 2005, Oyama reported that esophageal ESD achieved a 95% en bloc resection rate, 0% local recurrence rate, 0% perforation rate, and 6% mediastinal emphysema [16]. Furthermore, a multicenter retrospective cohort study of esophageal ESD for 373 lesions from 11 hospitals in Japan showed that the en bloc resection and R0 resection rates were 96.7% (95% CI 94.4–98.1%) and 84.5% (95% CI 80.5–87.8%), respectively. Meanwhile, perforation (including mediastinal emphysema), postoperative pneumonia, bleeding, and esophageal stricture occurred in 5.2% (95% CI 3.3–7.9%), 1.6% (95% CI 0.7–3.5%), 0%, and 7.1% (95% CI 4.9–10.2%) of patients, respectively [17]. Thus, high en bloc resection rates and the safety of esophageal ESD have been reported. Esophageal ESD is now widely performed as the main ER method for cT1a-EP/LPM plus cT1a-MM/cT1b-SM1 esophageal cancer.

Despite the popularity of ESD in Asian countries, ESD reports for BE cancer are limited because of its low incidence. A large-scale Japanese study [18] included 372 esophageal adenocarcinomas or esophagogastric cancer patients. Of 372 patients, 204 had SSBE and 34 had LSBE, whereas 122 had no underlying Barrett’s esophagus, and information was not available in 12 patients. Of 372 lesions, 321 were treated by ESD and 51 by EMR. En bloc resection and R0 resection rates were 99% and 88% for ESD and 61% and 49% for EMR, respectively. The local recurrence rate was analyzed in 316 patients (43 EMR and 273 ESD) who were followed without additional treatment. Local recurrence developed in six patients (14%) in the EMR group and one (0.4%) in the ESD group. The results of this study indicate that the recurrence of BE cancer, mainly originating from SSBE, is very low if the cancer is resected with R0 resection, even without ablation therapy. Following these favorable results of ESD, ESD rather than EMR is recommended as the ER method for BE cancer in Japanese guidelines [6].

### 3.2. EMR with a Cap-Fitted Panendoscope (EMRC)

EMRC is one of the EMR methods reported by Inoue in 1993 [19], in which a transparent cap is attached to the tip of the scope, into which the lesion is aspirated and then resected by the snare. For lesions smaller than 15 mm, EMRC has been reported to have an en bloc resection rate similar to that of ESD [20]. EMRC is considered a less physical burden for both patient and endoscopist because of having a shorter treatment time than ESD.

### 3.3. Two-Channel EMR Method

Two-channel EMR is one of the EMR methods developed by Momma in 1988 [21]. In this technique, the lesion is resected while grasping the lesion with forceps, thus enabling accurate treatment of a defined area with minimal tissue loss. The size of the mucosa that can be resected at one time is limited to approximately 25 mm. Local recurrence was observed in 16 (5.6%) of 287 patients with esophageal cancer after clinically complete resection [22].

### 3.4. Argon Plasma Coagulation (APC)

APC is a method in which a high-frequency current flows efficiently through ionized argon gas to inactivate and destroy tissue. It can safely and efficiently coagulate the mucosal surface layer and is used for hemostasis and tumor ablation. APC has been used to treat Barrett’s adenocarcinoma and early gastric cancer, and good outcomes have been reported [23,24,25]. The usefulness and safety of APC for endoscopically unresectable superficial esophageal neoplasia have been reported [26,27]. Although further prospective studies are necessary to confirm the usefulness of APC, it can be one of the treatment options for lesions that are difficult or unable to be resected via ESD due to the scar or the stenosis.

## 4. ESD in the West

In the European guidelines, ESD is also recommended for the resection of T1a esophageal SCC (ESCC) [28]. Since most of ESCC occurs in Asia, experience in ESD for ESCC is limited in the West. Thus, this technique has generally only been performed in expert centers [29]. A multicenter study of expert centers [29] showed that en bloc resection and complete resection for ESCC were 100% and 69.8%, respectively. Postprocedural bleeding, perforation, and stenosis occurred in 4.8%, 1.6%, and 23.8% of patients, respectively. Another report from an expert center showed an R0 resection rate of 96.7% and bleeding, perforation, and stenosis rates of 0%, 0%, and 11.5%, respectively [30]. Based on these reports from expert centers, ESD achieved a high R0 resection rate and acceptable adverse event rates. Further assessment is required if ESD is conducted in Western general hospitals in the future.

Regarding the results of ESD for BE adenocarcinoma. Chevaux et al. reported a retrospective study of ESD in 75 patients with BE cancer treated between 2007 and 2014. They performed ESD on lesions that were difficult to treat by conventional EMR, including multiple lesions, lesions larger than 15 mm, poor lifting, and suspected SM invasion. The median age of the patients was 68 years, and the median maximum diameter of treated lesions was 52.5 mm. The rate of en bloc resection by ESD was 90%, and the rates of curative resection of high-grade dysplasia and cancer were 85% and 64%, respectively. Regarding acute adverse events within 48 h after ESD, two cases of delayed bleeding and three cases of perforation were observed, both of which could be treated by endoscopic therapy [31]. Although these previous reports showed promising short-term results, a long-term, large-scale study is required to better understand ESD for BE cancer. In the West, EMR or ESD is followed by ablation to eliminate residual BE and reduce recurrent or metachronous BE cancers.

## 5. Complications

### 5.1. Intraprocedural Perforation

Unlike perforation in the stomach or colon, perforation during esophageal ESD may result in mediastinal emphysema or mediastinitis and, in severe cases, may cause a rapid deterioration in respiratory and circulatory status. Intraoperative perforation has been reported to occur in 0–6.9% of cases (Table 2), and most cases were successfully treated with clip closure [5,17,20,32] or polyglycolic acid (PGA) sheets and fibrin glue [33]. Early detection of intraoperative perforation and careful observation after ESD are vital to avoid surgical intervention [34].

### 5.2. Delayed Perforation

Delayed perforation is extremely rare with only a few cases after esophageal ESD being reported; in the five reported cases, the median time to onset of delayed perforation was six days. The mucosal defects were more than 1/2 circumference in all cases, and a local steroid injection was used in one case. In all cases, patients required invasive treatment, including sub-total esophagectomy, pleural drainage, or temporary stent placement [35,36,37,38].

### 5.3. Mediastinal Emphysema

Since the esophagus lacks a serous membrane, mediastinal emphysema can occur even without perforation, leading to subcutaneous emphysema in severe cases. In a report in which chest X-ray and chest CT were taken within 1 h after ESD to evaluate for mediastinal emphysema, 1.7% of chest X-rays and 31% of chest CT scans showed the presence of mediastinal emphysema [39]. Most mediastinal emphysemas detected by chest CT alone are minor and do not affect the clinical course. Therefore, there is no need to perform chest CT to evaluate mediastinal emphysema in patients without perforation. Esophageal ESD should always be performed with CO_2_, which is readily absorbed from the tissue, because air may cause severe mediastinal emphysema, even if only the muscle layer is exposed [40].

### 5.4. Delayed Bleeding

The frequency of delayed bleeding is low (approximately 1%), and careful prophylactic hemostasis is unnecessary. However, emergency endoscopic hemostasis should be performed immediately in the event of bleeding [41,42].

### 5.5. Postoperative Pneumonia

Pneumonia may result from aspiration of saliva or reflux during treatment. The frequency of postoperative pneumonia associated with esophageal ESD has been reported to be 1.6–2.6%. Since many patients have underlying diseases, such as emphysema, and are at high risk for severe pneumonia, any postoperative fever or poor oxygenation should be examined for the possibility of pneumonia [5,17]. ESD under general anesthesia may reduce the risk of aspiration pneumonia and should be considered an option for ESD.

### 5.6. Stricture

In previous reports, stricture occurred in more than 60% of post-esophageal ESD defects over 3/4 circumference and 100% of whole circumferential defects without any preventive methods [41]. The efficacy of preventive methods for stricture, including endoscopic balloon dilatation (EBD), local steroid injection, oral steroid administration, PGA sheets, cultured oral mucosal epithelial cell sheets, and stent placement, has been reported (Table 3). Previous reports have focused on the administration of steroids. Steroids are best suited as therapeutic agents for scar prevention because they suppress the inflammatory process and inhibit collagen synthesis and fibroblast proliferation. Previous systemic studies confirmed that steroid therapy can significantly decrease the stenosis rate and reduce the number of EBDs [43,44,45,46].

#### 5.6.1. Local Steroid Injections

Local steroid injections for non-whole circumferential mucosal defects showed a significantly lower stenosis rate of 10–45% than 61–82% for non-local steroid injections. In addition, the mean number of balloon dilatations required after stenosis tended to be lower for local injections (0–1.7 times with injections vs. 2–6 times without injections). Based on these results, the Japanese EMR/ESD guideline states: “Local injection of triamcinolone is weakly recommended when mucosal defects affecting ≥3/4 of the esophageal circumference occur after endoscopic resection for superficial esophageal SCC” [43,44,46,47]. However, the efficacy of local steroid injection after whole-circumferential lesions is insufficient, based on two studies that reported stenosis rates of 100% after local injection of triamcinolone.

#### 5.6.2. Oral Steroid Administration

Oral steroid administration has been reported as effective in preventing stenosis, with a 27–33% stenosis rate after whole-circumferential resection [48]. This rate is much lower than historical controls who did not receive oral steroid therapy. Oral steroid administration may be effective. However, there are concerns that treatment with steroids, especially at high oral doses, is associated with various adverse events. Reducing the dose of systemic steroids may reduce side effects but diminish their efficacy. The optimal dose of oral steroids should be further investigated. As for the whole-circumferential resection, 5 cm or more in axis length is considered a risk of stricture [14]. Accordingly, the guidelines state: “Endoscopic resection is weakly recommended for cT1a-EP/LPM superficial SCCs with a major axis length ≤50 mm and involving the entire circumference of the esophagus, upon implementing preventive measures for stenosis” [6].

#### 5.6.3. Innovative Strategies Using Tissue-Engineering Approaches

Tissue-engineering methods to prevent stenosis are divided into cell-based and scaffold-based therapies. In cell-based therapy, trophic effects of transplanted cells by releasing some substance such as cytokines are expected. Previous studies have shown that injecting keratinocytes [49] or adipose stromal cells [50] suppressed stricture in animal models. Ohki et al. transplanted epithelial cell sheets of oral mucosa to the esophageal ulcer, which enhanced the re-epithelialization of the ulcer and, thus, prevented stenosis in vivo [51]. In addition, autologous gastric mucosa [52], autologous esophageal mucosa [53], and autologous skin graft [54] are explored as possible grafts for the esophageal mucosal defect after ESD. In scaffold-based therapy, the growth of epithelial cells and the promotion of wound recovery are expected by extracellular matrix scaffolds. The efficacy of extracellular matrix scaffolds in preventing stricture is controversial and is still being evaluated in animal models and human patients [55,56]. Tissue-engineering approaches may provide feasible and promising solutions for post-ESD esophageal stenosis. However, these methods remain under investigation, and further research and large-scale clinical trials are warranted to confirm their safety and efficacy.

**Table 2:** Complication frequency of esophageal ESD.

Complications	Frequency	Reference
Perforation	0–6.9%	[5,16,17,20]
Mediastinal emphysema	31–63%	[39,57]
Delayed bleeding	0–0.7%	[41,42]
Postoperative pneumonia	1.6–2.6%	[5,17]
Stricture over 3/4 circumferential	over 60%	[41,58]
Stricture whole circumferential	100%	

**Table 3:** Stricture prevention methods and stricture rate. Data from [6,59] were summarized.

Stricture Prevention Method	Stricture Rate (%)			
	Over 3/4 Circumferential	Reference	Whole Circumferential	Reference
No treatment	63.8% (60/94)	[43,47,60,61,62]	100% (30/30)	[43,44,61,63]
Local steroid injection	15.9% (44/276)	[43,47,62,64]	100% (15/15)	[43,44]
Oral steroid administration	13.1% (16/122)	[60,61,62]	51.3% (19/37)	[61,63]
Local steroid injection + Oral steroid administration	12% (3/25)	[43]	57.1% (24/42)	[43,64]

## 6. Techniques

The esophagus has a narrow lumen, making it difficult to use gravity traction. In addition, as dissection proceeds, the lesion moves toward the anorectal side, making it challenging to maintain good traction and visual field. Therefore, esophageal ESD requires ingenuity to ensure safer treatment. Various methods have been devised to make ESD safer and more efficient.

### 6.1. Clip-with-Line Method

**Figure 1:**
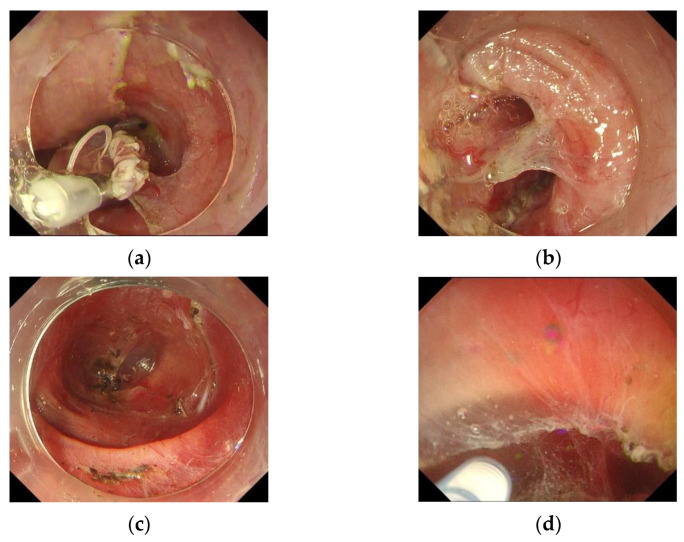
ESD techniques. (**a**,**b**) Clip-with-line method. (**c**) Tunnel method. (**d**) Underwater method.

The clip-with-line method was first reported by Oyama in 2002 and is widely used as a simple and effective traction method (Figure 1a,b). In a randomized controlled trial evaluating the use of threaded clips, treatment time was significantly shorter in the traction-assisted ESD (TA-ESD) group, with a median of 45 min in the TA group and 61 min in the conventional ESD group (*p* < 0.001). Intraoperative perforation was 4.3% in the conventional ESD group versus 0% in the TA-ESD group, indicating that the clip traction method with the thread also contributes to the safety of esophageal ESD [65,66]. Because there is no cost and safety risk increase, it is recommended to use Japanese ESD/EMR guidelines for esophageal cancer [6].

### 6.2. Tunnel Method

Conventional ESD for large lesions, especially those larger than two-thirds of the circumference, is time-consuming and carries a high risk of adverse events. The tunnel method for such large lesions has been reported to provide rapid dissection and a high R0 resection rate compared to conventional methods [67]. In a meta-analysis comparing the tunnel method versus the conventional method for esophageal neoplasms, the tunnel method was associated with a significantly higher en bloc resection rate (OR 3.98; 95% CI 1.74 to 9.12; *p* = 0.001), R0 rate (OR 2.29; 95% CI 1.54 to 3.46; *p* < 0.001), and rapid dissection (SMD = 1.52; 95% CI 1.09 to 0.83; *p* < 0.001) compared to conventional methods, with significantly lower complication rates, such as postoperative hemorrhage (OR 0.38; 95% CI 0.18 to 0.83; *p* = 0.02) and muscle layer injury (OR 0.44; 95% CI 0.28 to 0.70; *p* < 0.001), in the tunnel group [68].

### 6.3. Underwater ESD/Water Pressure ESD

The water pressure method uses the water delivery function from the knife to debride the submucosa while expanding the submucosal space and is reported to reduce complications in duodenal ESD by maintaining a good field of view in underwater immersion (Figure 1c,d) [69]. In esophageal ESD, underwater/water pressure ESD has also been reported to help maintain a good field of view [70,71]. Because of the risk of aspiration, endotracheal intubation or overtube is recommended if necessary.

## 7. Surveillance of Metachronous Esophageal SCC after ESD

After ER for esophageal cancer, endoscopic surveillance is recommended for the early detection of metachronous esophageal cancers. The presence of multiple iodine-unstained lesions in the esophageal mucosa is a risk factor for the development of metachronous esophageal SCC following endoscopic resection [72,73]. Metachronous cancer occurs annually in about 10% of patients with this condition [73]. A method to prevent metachronous SCC is alcohol abstinence, which significantly reduced the cumulative incidence of metachronous esophageal SCC in a prospective cohort study [73]. Another possible method is ablation using a radiofrequency ablation device [74,75], which may reduce the risk of metachronous SCC by eliminating multiple iodine-unstained areas [75]. However, further validation is required before introducing this method into our clinical practice. Even after such a preventive method, surveillance endoscopy is recommended for an extended period because metachronous esophageal SCC frequently develops, even after five years [76]. Although surveillance for metachronous esophageal SCC may improve the outcome of patients, no report examined the impact of different surveillance methods regarding surveillance interval on the early detection rate and mortality rate. In the guidelines of ER for esophageal cancer [6], a systematic review was conducted on how surveillance should be performed after ESD. In this systematic review, most reports indicated that endoscopic examinations were performed every 6–12 months as surveillance for metachronous esophageal SCC after ER. In addition, most of the detected cancers in these studies were successfully treated by endoscopic treatment. These reports recommend endoscopic examinations at least once a year in this guideline [6].

## 8. Expectations for Esophageal ESD

Future expectations regarding esophageal ESD include its widespread use worldwide and expanding its indication to larger cancers. Clinical benefits of ESD, such as high en bloc resection rate and low local recurrence rate, are already recognized widely. Although previous ingenious attempts, such as the clip-with-line method or tunnel method, improved the feasibility of ESD, it remains a challenging procedure since complication of esophageal ESD sometimes results in severe consequences. Further innovative attempts or development of new devices, endo-knife, or ESD devices are recommended for widespread use of this method worldwide. Indications of ESD in terms of the technical aspect, oncological aspect, and risk of adverse events should be considered. ESD for whole-circumferential lesions is a controversial topic. ESD for such lesions is sometimes delayed considering the risk of adverse events, especially refractory stenosis, after ESD. Refractory stenosis will be overcome in the future as various approaches are developed for this issue. Meanwhile, we can apply the modified ESD method, stepwise ESD [77], for whole-circumferential lesions. In this method, two-thirds of the circumference is resected by ESD with triamcinolone injection to prevent stenosis, followed by ESD for a remnant lesion with triamcinolone injection 2–5 months later. An excellent result, only one stenosis case out of three patients, was reported after this method. Although there is a minor concern from the oncological aspect regarding the risk of local recurrence derived from piecemeal resection, this method may be a good alternative to en bloc ESD for whole-circumferential lesions.

## 9. Future Perspectives

ESD is a curative treatment only for cancers with an ignorable risk of metastasis. Indication of ESD is limited by the safety of ER and by the risk of metastasis for more advanced lesions, e.g., T1b or T2 cancers. Future research should be aimed to develop some technique to resect such advanced lesions and to concur the risk of metastasis. From the technical aspect, several cases of endoscopic full-thickness resection (EFTR) for a submucosal tumor in the esophagus have been reported [78,79]. Given that EFTR is a modified ESD or peroral endoscopic myotomy (POEM), the technique itself is not so difficult for endoscopists who have enough expertise on ESD or POEM. If EFTR for esophageal cancer becomes technically feasible, local complete resection of the primary tumor could be achieved, even for T1b or T2 cancers. From the oncological aspect, T1b or T2 cancers have a considerable risk of metastasis, and the risk should be treated adequately. Sentinel node navigation surgery (SNNS) has been used as a minimally invasive surgery for breast cancer and melanoma [80,81,82]. Similarly, based on sentinel node theory, additional lymph node dissection could be skipped in esophageal cancer patients with a negative sentinel node (SN) biopsy. SNNS, combined with EFTR, is expected to be an extremely minimally invasive treatment in the future. In addition, the effectiveness of ER followed by CRT for T1 cancer has been reported [83]. If complete local resection of T1b and T2 cancer can be achieved with EFTR, combining additional CRT based on pathological diagnosis may become a less invasive alternative to esophagectomy for these cancers. Moreover, the recent development of genomic medicine accelerated the introduction of precision medicine in our practice. Circulating tumor DNA (ctDNA) analysis is a promising strategy to detect evidence of minimal residual disease that could ultimately be the source of later metastasis. A clinical trial showed the potential of a ctDNA-guided approach to the treatment after colon cancer surgery [84]. If this strategy progresses, endoscopic resection of T1b or T2 cancer combined with a ctDNA-guided approach would be a promising strategy for these cancers.

## Figures and Tables

**Table 1:** ESD indications for SCC.

Clinical T1a EP/LPM noncircumferential lesion
Clinical T1a EP/LPM circumferential lesion ≤ 50 mm
Clinical T1a-MM/T1b-SM1(invading submucosa ≤ 200 μm) cancer non-circumferential lesion

## Data Availability

Not applicable.
